# The Sandwich Generation Diner: Development of a Web-Based Health Intervention for Intergenerational Caregivers

**DOI:** 10.2196/resprot.5488

**Published:** 2016-06-06

**Authors:** Ann M Steffen, Joel Epstein, Nika George, Megan MacDougall

**Affiliations:** ^1^ Department of Psychological Sciences University of Missouri-St. Louis St. Louis, MO United States; ^2^ Missouri Institute of Mental Health University of Missouri-St. Louis St. Louis, MO United States; ^3^ St. Louis VA Medical Center St. Louis, MO United States

**Keywords:** intergenerational relations, health education, caregivers, intervention studies, Internet

## Abstract

**Background:**

Women are disproportionately likely to assist aging family members; approximately 53 million in the United States are involved with the health care of aging parents, in-laws, or other relatives. The busy schedules of “sandwich generation” women who care for older relatives require accessible and flexible health education, including Web-based approaches.

**Objective:**

This paper describes the development and implementation of a Web-based health education intervention, The Sandwich Generation Diner, as a tool for intergenerational caregivers of older adults with physical and cognitive impairments.

**Methods:**

We used Bartholomew’s Intervention Mapping (IM) process to develop our theory-based health education program. Bandura’s (1997) self-efficacy theory provided the overarching theoretical model.

**Results:**

The Sandwich Generation Diner website features four modules that address specific health care concerns. Our research involves randomly assigning caregiver participants to one of two experimental conditions that are identical in the type of information provided, but vary significantly in the presentation. In addition to structured Web-based assessments, specific website usage data are recorded.

**Conclusions:**

The Sandwich Generation Diner was developed to address some of the informational and self-efficacy needs of intergenerational female caregivers. The next step is to demonstrate that this intervention is: (1) attractive and effective with families assisting older adults, and (2) feasible to embed within routine home health services for older adults.

## Introduction

### Background

Researchers are faced with minimal operational guidance when attempting to develop complex interventions to reduce the gap between clinical evidence and behavioral practice [1]. There is, however, a growing recognition that behavioral-health interventions should be grounded upon a solid theoretical framework [2]. Not only are theory-based interventions more likely to be effective, they can also provide an understanding of causal pathways for change [2]. Unfortunately, although there are numerous behavioral-health theories to draw upon, it is challenging to translate these theories into practical interventions [2,3].

In an effort to address these issues, Bartholomew [4] developed an Intervention Mapping (IM) protocol that provides a process for developing theory-based health education programs. In IM, researchers use these six iterative and reciprocal tasks to create their interventions: (1) assessing the problem, (2) specifying program objectives, (3) identifying theory- and evidence-based behavior change methods, (4) creating the intervention, (5) planning the implementation, and (6) planning the evaluation [5]. In this article, we describe the development of our *Sandwich Generation Diner* intervention for intergenerational caregivers within the context of the IM framework.

### Intervention Mapping Step 1: Assessing the Problem

#### Prevalence and Correlates of Medication Nonadherence in Older Adults

Older adults have more chronic medical conditions and a corresponding higher rate of medication use, spending three times the national average for prescription drugs [6]. Although some do an excellent job of managing their medications, a significant number have difficulty with this task. Medication mismanagement and errors may have dire consequences for seniors, including a higher risk for falls, delirium, excess disability, hospitalizations, and death [7].

#### The Role of Family Involvement in Medication-Related Behaviors

Many older adults have close reciprocal relationships with younger members of their family, and due to physical or cognitive limitations, receive assistance with health care visits and medication management. There are an estimated 3.3 million older adults in the United States who receive assistance with both physician visits and prescribed medications, and these are a high-need group [8]. More than 75% of this population require aid with mobility, self-care, or household activities, and approximately 60% have possible or probable dementia. Involvement by a family care partner typically persists over time, and is more likely to be a younger female family member rather than a spouse [8]. Women make approximately 80% of health care decisions for their families and are more likely than men to be the caregiver when an older family member is ill [9]. Although caring for an older relative may have negative impacts on women’s mental and physical health, caregivers often view their role as deeply meaningful [10].

#### Health Education for Intergenerational Caregivers

Few community-based health education programs offer support explicitly targeting intergenerational caregivers [10-12]. Those that are available have significant limitations. Community-based programs are often inaccessible or burdensome due to the care recipient’s need for ongoing supervision. In-home programs can affect a number of care outcomes, but also include challenges such as the cost of individualized services and staff travel. Psychoeducational groups may be helpful, but they require participants to travel to the program. Consequently, the “sandwich generation” women who care for older relatives require more accessible and flexible health education approaches than is generally available.

### Intervention Mapping Step 2: Specifying Program Objectives

#### Web-Based Health Education

Technological advances have created new opportunities for reaching consumers through Web-based and other distance-based health education and support [13-15]. The Internet has become an important source of health information. Eighty percent of internet users, and 59% of the US population, look on the Internet for health information [16]. The proportion of adults aged 65 and older who use the Internet to search for information has grown to 53%; of these, 70% report using the Internet daily [17]. As providers continue to adopt eHealth technologies, the range of activities for consumers to participate in their health care continues to expand and includes everything from engaging in condition-specific discussions to entering information in personal health records [18]. Not only does participating in Web-based health care programs provide clear benefits in terms of wellness, it can also result in increased motivation and self-efficacy for managing care [19].

#### Receipt of Grant Award

Our team has had a long history of developing eHealth interventions including an interactive public education exhibit, continuing education courseware, and smartphone apps [20,21]. These efforts, along with our experience developing video-based interventions for dementia caregivers [14,15,22] led to an award from the authors’ university.

The primary aims for our project were to: (1) increase caregivers’ knowledge and use of effective medication management strategies, (2) decrease medication-related hassles perceived by women caring for an older relative, and (3) decrease rates of adverse medication-related events for older care recipients. We assembled a diverse work group from units on campus (ie, Psychological Sciences, Gerontology, and the Missouri Institute of Mental Health) as well as a community advisory board and an interdisciplinary panel of consultants to assist with study content and dissemination.

## Methods

### Intervention Mapping Step 3: Identifying Theory- and Evidence-Based Behavior Change Methods

In their meta-analysis of the features of effective Web-based health promotion interventions, Lustria et al [13] describe the importance of grounding programs in empirically supported theories of change. Davis and colleagues [2] argue that many of these theories provide little guidance regarding how to translate underlying concepts into practical interventions. Bandura’s Social Cognitive Theory (SCT) [23] is a notable exception. Bandura states that learning occurs in a social context and is shaped by reciprocal relationships between the person, environment, and behavior. SCT involves a heavily agentic perspective, in which individuals are seen as goal-directed and exert intentional influence over their functioning; these intentional actions then impact the course of events. A number of principles are incorporated within this theory, including reciprocal determinism (interaction of person, environment, and behavior), behavioral capacity (actual ability to perform a behavior through knowledge and skills), observational learning, reinforcements, outcome expectations, and self-efficacy [23]. The construct of self-efficacy is an important facet of SCT, and refers to beliefs in one’s ability to execute courses of actions to manage situations important within a specific life domain. In Bandura’s theory, self-efficacy beliefs influence whether coping efforts will be initiated, how much effort will be invested, how long effort is sustained in the face of aversive experiences and obstacles, and affect vulnerability to emotional distress. Self-efficacy beliefs can be shaped by various means including mastery experiences, social observation, and social persuasion [24].

In the area of aging, the model has been applied to understanding dementia family caregiving [25] and medication adherence [26]. When individuals face health-related caregiving challenges, those with low self-efficacy focus on negative aspects of the situation (eg, personal deficiencies, task difficulties). In essence, they “lose heart.” This focus on negative cognitions reduces motivation to initiate an activity, impacts task persistence, and leads to negative affective states, which then perpetuates the cycle [27].

Because Bandura argues that self-efficacy can be improved via social observation, many interventions attempt to promote behavior change by increasing self-efficacy through observational learning of similar others. These interventions often use dramatic enactments of scenarios that the target audience may face in their daily lives. By identifying with and watching others successfully master challenges, viewers gain an improved sense of their own mastery. Using this model, researchers have successfully used serial radio dramas to improve human immunodeficiency virus outcomes [28,29]. Our own work has demonstrated the benefits of video-based stories for improving knowledge of substance abuse [30].

Based on this prior validation, we chose to ground our current intervention on Bandura’s SCT, with a particular focus on influencing the caregiving self-efficacy beliefs of intergenerational caregivers. We incorporated a number of elements of SCT, while aiming to: (1) acknowledge the importance of social context by distinguishing among skills needed in different caregiving situations, (2) influence behavioral capacity through instruction in specific health care management and interpersonal communication skills, (3) foster observational learning through serial video narratives of caregivers, and (4) enhance self-efficacy beliefs by developing story lines that show characters mastering progressively more challenging situations. We hypothesized that individuals who viewed true-to-life vignettes of individuals successfully overcoming difficulties providing care for their elderly family members would demonstrate improved outcomes as compared with individuals who received information presented in a more didactic format.

### Intervention Mapping Step 4: Creating the Intervention

#### Content Outlines

We began our development efforts by creating detailed content outlines of the information we wanted to convey to our target audience. We had a team of expert consultants including pharmacists and nurse practitioners, as well as social work, psychology, and public health professionals consult with us about the most common difficulties associated with health care and medication management in older adults. We cross-referenced these content areas with existing behavioral health literature regarding best practice guidelines. We then included the overlapping recommendations as final content to be included in the intervention script, making sure to couch them within the context of social cognitive theory (eg, selecting situations identified as common challenges, developing the vignettes to show an increase in challenge/difficulty over the narrative, linking the behaviors of the characters to specific skills, having the characters demonstrate mastery at the end). This process resulted in the creation of four subject areas: (1) medication management, (2) recognizing and responding to alterations in cognition (delirium and dementia), (3) managing health care appointments and communication with providers, and (4) communication and planning strategies for family members.

#### Development of Creative Approach

With the content clearly articulated, we were ready to develop the overarching creative approach for our intervention. In past work, we discovered that developing an underlying story or metaphor to convey our content helped to increase participant engagement. For example, in one of our early educational programs designed to teach students about the biology of addiction, we embedded our learning objectives within a narrative story about aliens coming to Earth to learn how to play basketball [31].

For the current intervention, we needed to develop a creative approach that would not only engage our target audience (middle-aged women caring for elderly family members), but would also reflect a diverse set of demographic characteristics. During a series of brainstorming sessions, we finally settled upon the idea of our intervention taking place in a diner with a wise and seasoned waitress serving as the primary character. Through interactions with her various customers, viewers would be able to see various health care challenges being overcome.

#### Script Writing

It was only after the content and creative approach were finalized that we were able to begin writing the intervention’s scripts. For each of the four content modules, we structured the story to evolve over the course of five separate vignettes. Each story began with the waitress character introducing the primary dilemma the characters were facing. Then, in each subsequent vignette, the characters demonstrated a growing sense of efficacy in their ability to solve the problems they were having while caring for their family members. In order to increase the likelihood of participants watching the vignettes, each is less than 4-minutes long.

#### Review of Intervention Plans

When the scripts for each module were completed, we engaged in a second round of expert consultation. Once again, we used our interdisciplinary approach to discuss the relevance, use, and goals of this portion of our intervention. After completing suggested modifications to the script, we presented it to a small group of selected “beta testers.” These individuals were lay persons unaffiliated with the intervention who we asked to review the scripts for readability and clarity of the dialogue. We incorporated this group’s suggestions into the final script.

#### Content Acquisition

After completing the scripts, we then created detailed storyboards for our Web-based intervention. Following these specifications, we produced custom graphics for the program’s logo and interface. In this way, we were able to “brand” our intervention with an engaging appearance and name – “Sandwich Generation Diner.” We used these graphics for all materials associated with the intervention (eg, marketing pieces, downloadable files, etc).

The scripts themselves called for a number of characters across a broad set of demographic characteristics. Through advertisements on local actor’s message boards, word of mouth, and notes to previous actors we had worked with in the past, we assembled a pool of candidates to appear in our production. We then used a series of phone and live auditions to settle upon the final cast.

In order to improve the authenticity of our production, we placed a special emphasis on finding the perfect location at which to shoot our videos. We eventually arranged with the management of a local diner and were able to use their space for eight, after-hours sessions during the course of a single month. In addition to providing us authentic props, the management also cooked us the food that was specified in the script.

We asked the actors to memorize their lines; prior to shooting each scene, we had them run their lines in place. This procedure reduced rehearsal time and facilitated the production process. For each scene, we recorded both wide and close-up shots. Although this process required the actors to deliver their lines multiple times, it also provided us with multiple options for editing the final footage.

#### Coding

Because we wanted to roll out our intervention to participants across the course of a month, we needed to develop a solution that allowed for flexibility in the way our content would be displayed and delivered. In the end we chose to create a WordPress site and assigned all participants a unique login. By using the s2Member plug-in, we were able to code the site in such a way that it would modify its display according to the credentials we assigned each user. This procedure allowed us to set user’s access levels in terms of being part of the control or experimental condition and how far along they were in the intervention protocol. Each week, we increased the participants’ access level so that they would automatically see subsequent portions of the intervention.

## Results

### Program Description

Regardless of experimental condition to which they were assigned, all participants log onto our site and are automatically presented with the appropriate content based on their user credentials. By manipulating user’s access levels in the website’s control panel, we have been able to sequentially roll out the intervention across 4 weeks (Phase I) and test impact of accessing all intervention materials at once (Phase II). To encourage viewing of our materials, we send weekly reminder emails to the participants.

The overall appearance of our site is largely the same for participants in both the Narrative Vignette and Comparison Didactic conditions. See Multimedia Appendix 1 for a screen shot of our site’s main menu. After clicking on one of the primary content areas, participants are presented one of the submenus illustrated in Multimedia Appendix 2. Although participants in both groups have access to the didactic portable document format (PDF) informational sheets (see Multimedia Appendix 3) and talking head expert videos (see Multimedia Appendix 4) presented in the left column, only participants in the Narrative condition have access to the scripted vignettes (described above and illustrated in Multimedia Appendix 5). Although all links are visible throughout the entire intervention period, we sequentially enable them throughout the course of an individual participant’s 4-week trial. Participants are free to choose to view the information in any order and spend as much or little time on the site as they would like.

### Health Education Content

In keeping with the self-efficacy literature, we developed storylines consistent with common situations and needs of intergenerational female caregivers. Actors were selected who represented our target demographics for this study.

#### Managing Medications

This content area includes a discussion of adverse events linked to medications (eg, falls, delirium, nursing home placement, negative health outcomes), and provides information about the most effective ways to manage scheduling of doses and refills. This module also includes psychoeducation about the role a pharmacist can play in medication management problem solving, as well as a list of basic and advanced questions that can be used to help improve medication management and organizational skills. The module also provides further information about the benefits of assessing interaction effects of over-the-counter medications with prescriptions that the care recipient already takes. The module highly encourages consultation with pharmacy staff.

#### Signs of Confusion

Given that caregivers of older adults with undiagnosed cognitive impairment may not know how thinking abilities relate to medication management, this content area provides participants with information about the signs and symptoms of delirium and dementia, as well as ways to distinguish the two. Caregivers are given information about the high rates of undiagnosed delirium and dementia in community-dwelling older adults and the benefits of medical evaluation and diagnosis. The module also presents information about the process for obtaining a diagnosis for a progressively dementing neurocognitive illness, including material about brief cognitive screens and neuropsychological testing.

#### Health Care Visits

This content area provides caregivers with information about how to prepare for health care visits that they attend with their loved one. Caregivers are given a list of example questions they can ask medical providers about current medications, newly prescribed medications, over the counter medications, and side-effects. This module also addresses legal and Health Insurance Portability and Accountability Act (HIPAA) barriers to full communication between family members and health care professionals. The content area explains the need for signed releases on file in the patient’s chart to allow these conversations and provides an example of a nationally available form for durable power of attorney for health care, named “Five Wishes.”

#### Talking Together

This content area focuses on developing communication skills between the caregiver and care recipient. Caregivers are given instruction on how to use basic communication strategies such as eye contact, tone, and “I” language. This module also provides examples of medication-related conflict between the caregiver and care recipient, and offers suggestions for effective communication strategies when negotiating these disagreements. In addition, caregivers are encouraged to use these communication skills when discussing potentially conflictual topics such as housing, financial planning, driving, and health care plans. This module also addresses legal and HIPAA requirements for full communication between family members and health care professionals.

### Intervention Mapping Step 5: Planning the Implementation

Our goal is to demonstrate that this intervention is (1) attractive and effective with families assisting older adults enrolled in home health care (a medically vulnerable population), and is (2) feasible to embed within routine home health services for older adults. We aim to evaluate the intervention’s impact on family–clinician communication and reported caregiving self-efficacy and role overload. The original funding source (an internal grant from the investigators’ university) supported the development of the Web-based intervention and collection of pilot data.

### Intervention Mapping Step 6: Planning the Evaluation

We first developed a preliminary evaluation with a community sample. Initial pilot data supporting the intervention will be valuable in developing collaborative relationships with health care agencies and systems serving older adults.

### Participants

Adult women providing informal care to an older adult are recruited through print advertisements placed in health care and community locations throughout a large Midwestern city, and through study announcements posted on social media. Interested individuals are directed to a brief Web-based screening survey.

### Intervention Procedure

We email eligible participants who consent to the study a longer preintervention survey. Upon completion of the preintervention survey, we randomly assign participants to either the Narrative Vignette or Comparison Didactic condition. We use block randomization to balance dementia caregivers and nondementia caregivers across study conditions. After randomization, we email participants with their unique login and password along with instructions for using the website. We also instruct them not to allow anyone else to log in to the website using their credentials.

During the 4-week intervention period, we send participants weekly emails. At the end of their trial, we send participants a postintervention assessment survey, and a 1-month follow-up survey. We then conduct a brief phone interview to collect qualitative data. For participants who had been in the Comparison Didactic condition, we then allow them to have access to the narrative vignettes. Finally, 6 months after completing the intervention, we invite participants to complete another brief follow-up Web-based assessment.

Participants complete Web-based assessments at preintervention, postintervention, 1-month follow-up, and 6-month follow-up. We collect descriptive information about the participant’s caregiving situation and demographic information about the caregiver and care recipient. Measures include medication risk [32], falls, and the Centers for Disease Control Healthy Days for the care recipient [33], caregiving medication administration hassles [34], role overload [35], self-efficacy beliefs [25], and family–health care provider communication [36]. We gather qualitative data about project feedback via a brief phone interview 1 month after completing the intervention. Participants receive US$25 gift cards after completing each of the assessments. Participant’s website usage is automatically tracked including information about the dates and times of website use and any downloads of Web-based material over the study period.

We are presently in the data collection phase of this process, and initial participant characteristics and satisfaction ratings are promising. Caregiver participants (N=137) are ethnically diverse at 31% (43/137) non-White compared with 26% of US residents being non-White. Participants predominantly care for a frail parent, with 85% (117/137) being a daughter or daughter-in-law of the older adult. Caregivers engaged in significant medical management tasks for older adults with a number of chronic and comorbid health conditions. Participants reported satisfaction with the intervention (mean=5.65, standard deviation= .97) on a 7-point scale with a score of 7 being most satisfied.

## Discussion

### Summary

We developed and are currently evaluating an eHealth intervention (in a randomized trial format) for female intergenerational family caregivers actively involved with the health care of an older relative. Both conditions are designed to improve medication management and related health care behaviors in informal caregivers of older persons with physical and/or cognitive impairments. The didactic comparison condition contains a series of downloadable PDF “Handouts” with information about managing medications, attending a health care visit, causes of confusion in older adults, and communication with the older family member. Each section includes one video of an expert providing information in traditional didactic voice. The narrative vignette condition includes the same content as above, with additional serial Web episode storylines that show caregivers interacting with their loved one, and problem solving concerns in each of the aforementioned domains.

### Program Strengths

Strengths of our program include a solid conceptual grounding of the intervention within self-efficacy theory [27], and creation of a flexible Web-based format that allows caregivers to explore health education content areas of most interest to them. We also believe that targeting intergenerational caregivers involved in medication management for older adults is novel and justified, given the literature describing differences between spousal caregivers and adult children in levels of frustration with these types of tasks [37]. Many studies show that caregivers have increased time constraints when compared with similar noncaregiving individuals [10]; the Web-based availability of our intervention materials may decrease the time investment necessary for caregivers to obtain aid [12].

### Limitations and Lessons Learned

Because this study occurs over a relatively brief period (1 month), future research may benefit by extending the amount of follow-up contact and assessment points. This study has not implemented specific strategies to improve access for caregivers who are lower income or who demonstrate lower educational attainment. Such individuals generally have limited access to technological resources or may show lower general engagement with health care and health-related interventions. Future studies may explore the current barriers to technological access and eliminate these by providing other avenues to Internet resources (ie, library cards or rented tablets).

### Conclusions

The *Sandwich Generation Diner* has been developed to address some of the informational and self-efficacy needs of intergenerational women caregivers. Within the IM framework, development of the *Sandwich Generation Diner* intervention involved: (1) assessing the problem, (2) specifying program objectives, (3) selecting an overarching theoretical model, (4) creating the intervention, (5) implementing the intervention, and (6) evaluating the intervention. Bandura’s self-efficacy theory [27] provided the overarching theoretical model. The integration of serial narrative vignettes with multimedia resources is intended to result in a rich and meaningful intervention. We look forward to completion of the ongoing efficacy trial to evaluate the extent that we have accomplished these aims.

## Appendix

Multimedia Appendix 1

Multimedia Appendix 2

Multimedia Appendix 3

Multimedia Appendix 4

Multimedia Appendix 5

## Abbreviations

HIPPA: Health Insurance Portability and Accountability Act
IM: intervention mapping
PDF: portable document format
SCT: social cognitive theory
