# Supplementary figures and images for: The Sandwich Generation Diner: Development of a Web-Based Health Intervention for Intergenerational Caregivers
Source: JMIR Res Protoc. 2016 Jun 6;5(2):e91. doi: 10.2196/resprot.5488 (PMC4914775; doi:10.2196/resprot.5488)

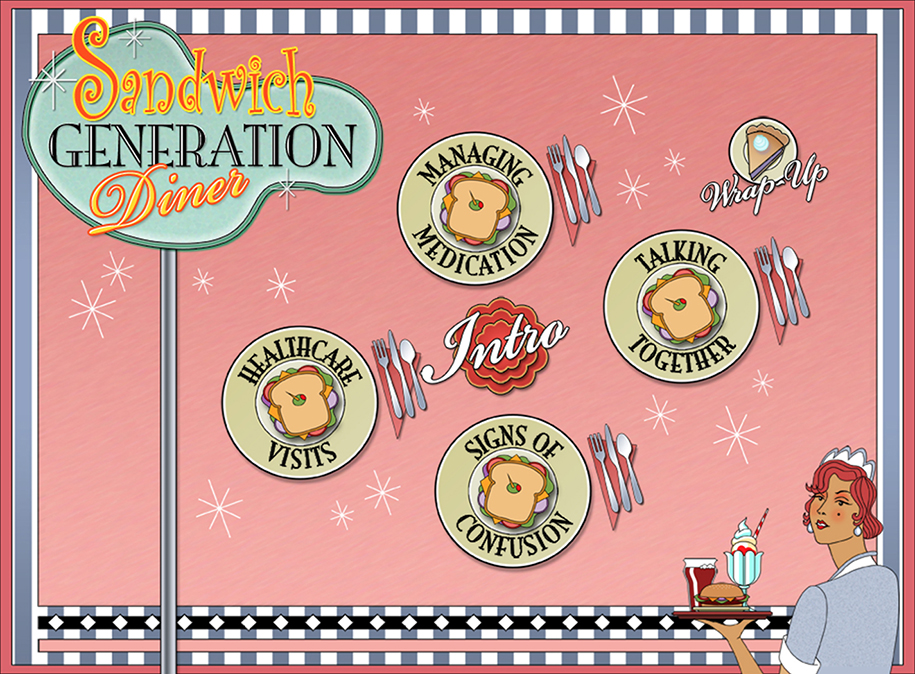

Supplement: Multimedia Appendix 1 [file resprot_v5i2e91_app1.png]

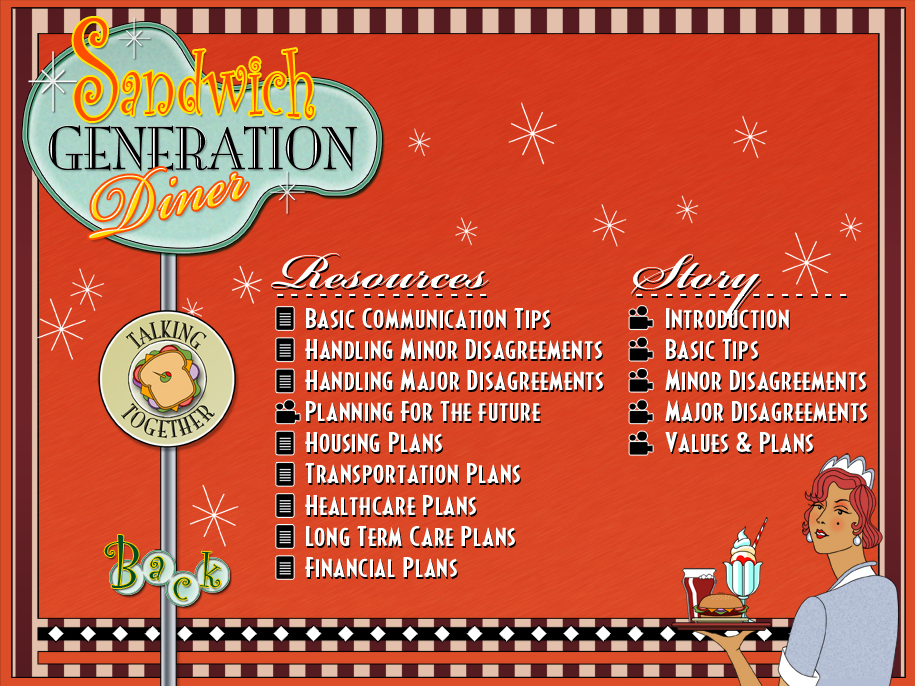

Supplement: Multimedia Appendix 2 [file resprot_v5i2e91_app2.png]
