# Supplementary material for: The Sandwich Generation Diner: Development of a Web-Based Health Intervention for Intergenerational Caregivers
Source: JMIR Res Protoc. 2016 Jun 6;5(2):e91. doi: 10.2196/resprot.5488 (PMC4914775; doi:10.2196/resprot.5488)
Supplement: Multimedia Appendix 3 [file resprot_v5i2e91_app3.pdf]

Sandwich GENERATION Diner

Basic Communication Tips

Talking with family and friends about health concerns may sound easy, but can be surprisingly difficult. This list of basic tips and strategies can be used to help families talk about healthcare needs.

- Get rid of Distractions- turn off the T.V., sit together in a quiet room.
- Do not interrupt the other individual.
- Actively Listen – summarize what the other person said. Ask if you are really understanding what is most important to them.
- Accept the person's concerns as valid. For example: "I understand why you are worried - that makes sense from your point of view. Let's talk about some choices that respect your wishes."
- Communicate your points using "I" statements and non-accusatory language.

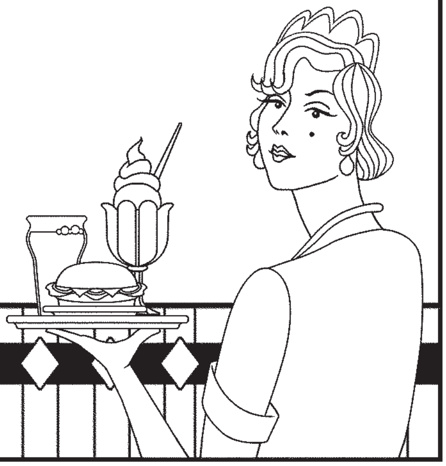

University of Missouri – Saint Louis • Department of Psychology • caregiverpsychology@umsl.edu

Basic Communication Tips (continued)

- Make eye contact and call the person by name.
- Be aware of your tone and how loud your voice is.
- Think about your body language. What faces are you making? Do these faces send the caring message you want?
- Compromise. Each person should feel as though they made some part of the decision.
- Be direct, specific and positive in taking about life values and goals.
- Ask each other about personal values. What is most important? For example, "Do you think it is important to follow the doctor's instructions?" "Why or Why Not?"

Sandwich GENERATION Diner

University of Missouri – Saint Louis • Department of Psychology • caregiverpsychology@umsl.edu
